# Supplementary material for: Scalable Functionalization of Polyaniline-Grafted rGO Field-Effect Transistors for a Highly Sensitive Enzymatic Acetylcholine Biosensor
Source: Biosensors (Basel). 2022 Apr 27;12(5):279. doi: 10.3390/bios12050279 (PMC9138234; doi:10.3390/bios12050279)
Supplement: Supplementary file 1 [file biosensors-12-00279-s001.zip › biosensors-1699380-supplementary.pdf]

biosensors

Supplementary Materials

Scalable Functionalization of Polyaniline-Grafted rGO Field-Effect Transistors for a Highly Sensitive Enzymatic Acetylcholine Biosensor

Dongsung Park [1,2,†], Dongtak Lee [1,†], Hye Jin Kim [3], Dae Sung Yoon [1,*] and Kyo Seon Hwang [2,*]

[1] School of Biomedical Engineering, Korea University, Seoul 02841, Korea; dpark7047@gmail.com (D.P.); ehdxor11@korea.ac.kr (D.L.)
[2] Department of Clinical Pharmacology and Therapeutics, College of Medicine, Kyung Hee University, Seoul 02447, Korea
[3] Institute of Chemical Process (ICP), Seoul National University (SNU), Seoul 08826, Korea; hyejinkim.mail@gmail.com
* Correspondence: dsyoon@korea.ac.kr (D.S.Y.); k.hwang@khu.ac.kr (K.S.H.)
† These authors contributed equally to this work.

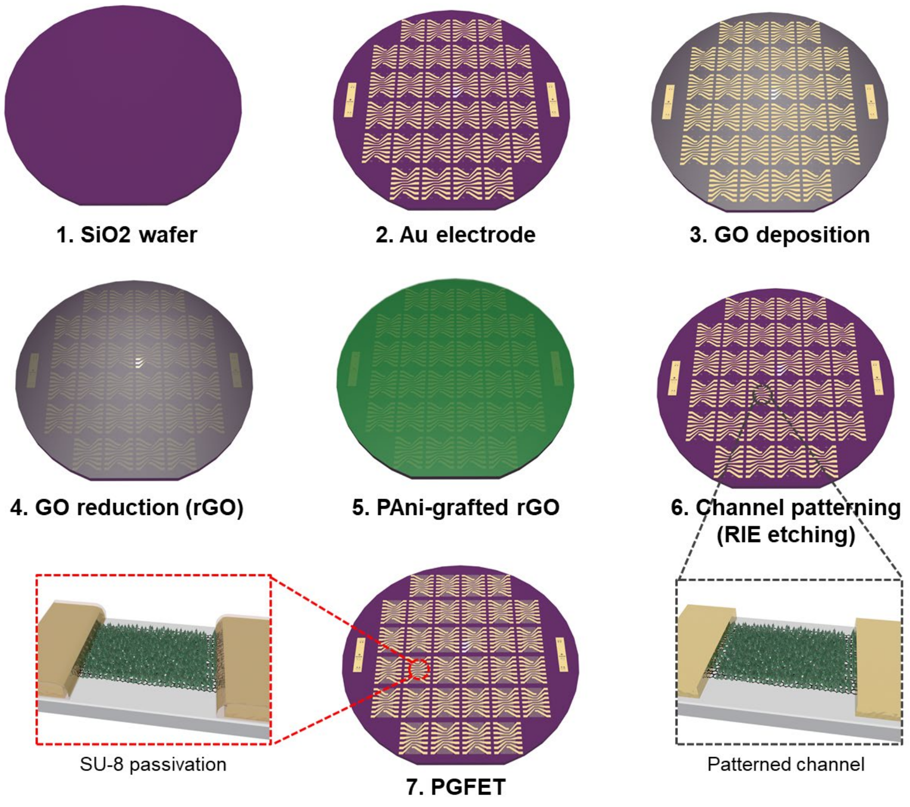

Figure S1. The wafer-scale fabrication procedure of PGFET**.**

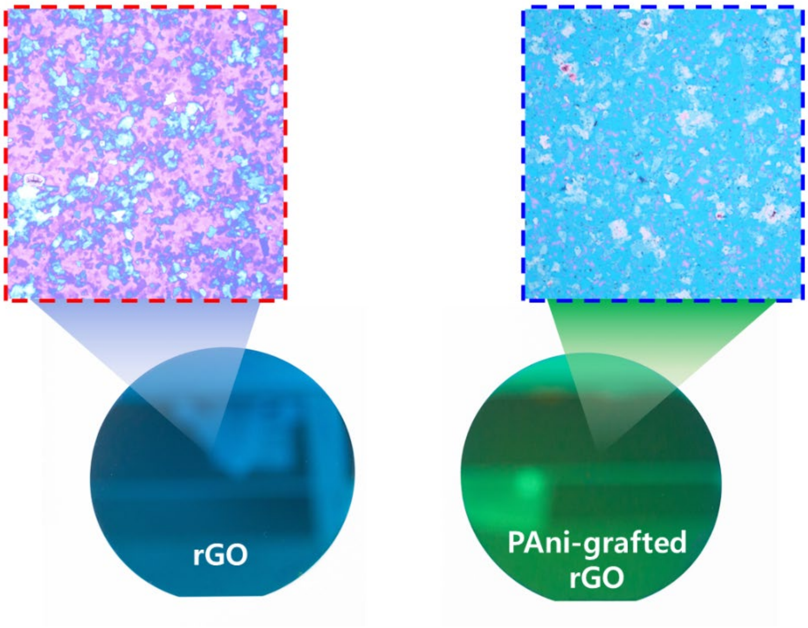

Figure S2. The photographic images of rGO and PAni-grafted rGO deposited on 4 in. wafers. The magnified images of rGO (red) and PAni-grafted rGO showing completely coverd the rGO surface.

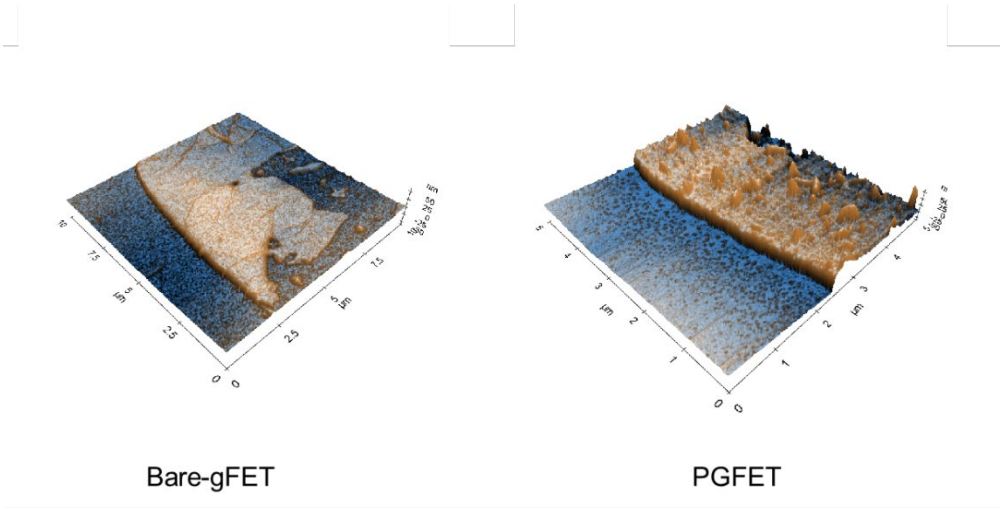

Figure S3. Three-dimensional AFM images of bare-gFET and PGFET.

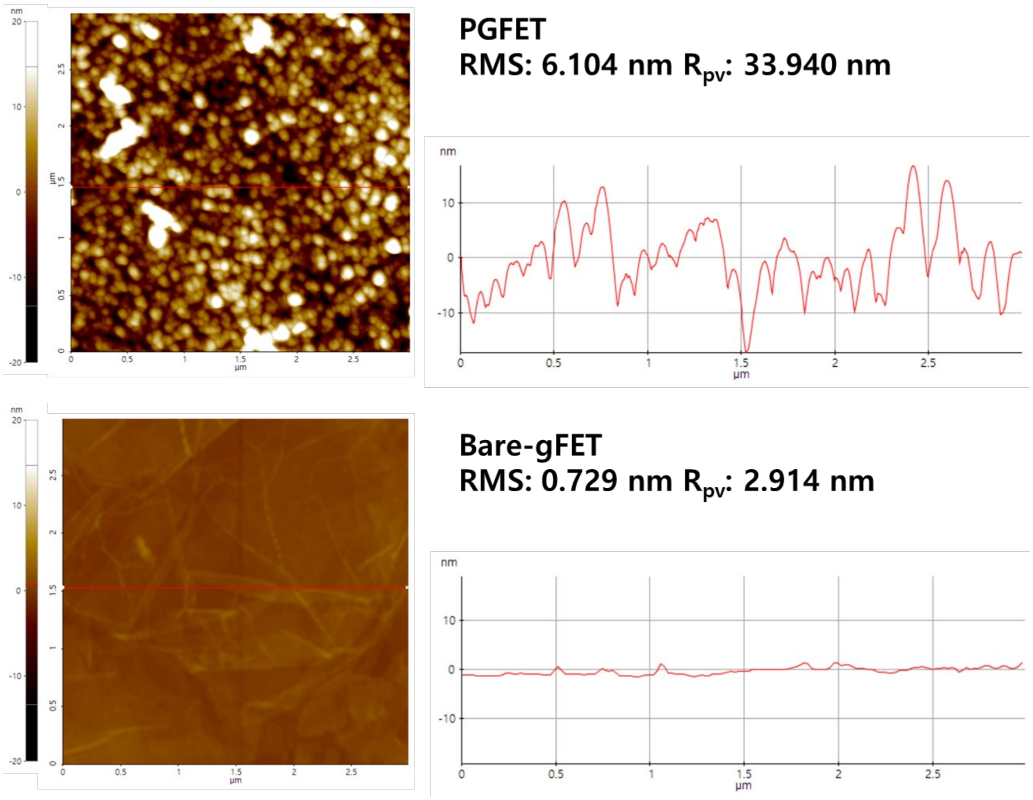

Figure S4. Comparison of morphologies of PGFET and bare-gFET. AFM image and surface roughness of PGFET and bare-gFET. (RMS, rms surface roughness, R_{pv}, peak-to-valley roughness).

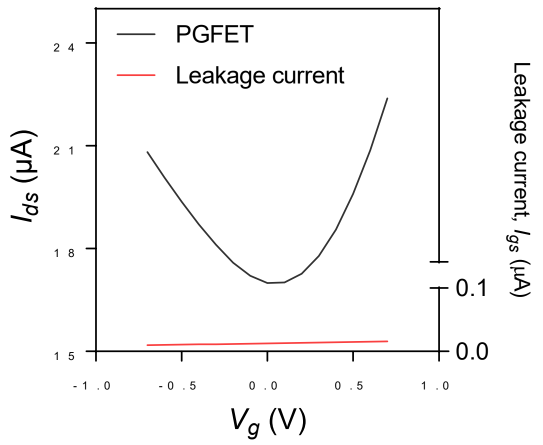

Figure S5. Comparison between leakage current and the source-drain current of PGFET with varying the gate voltage (Vg), representing that leakage current is negligible (~27 nA).

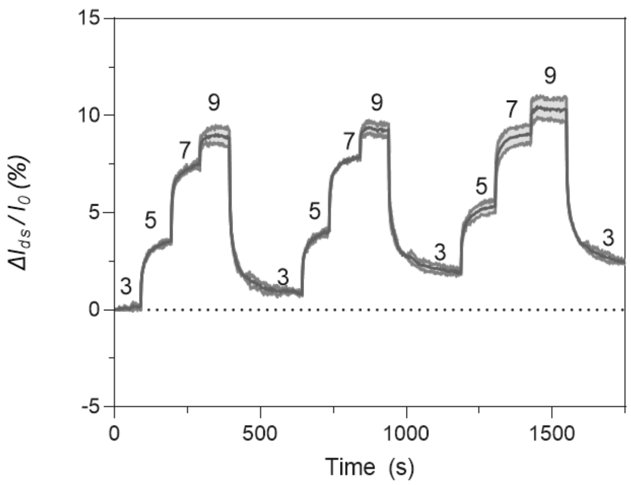

Figure S6. The real-time output responses of bare-gFETs for three pH cycles.

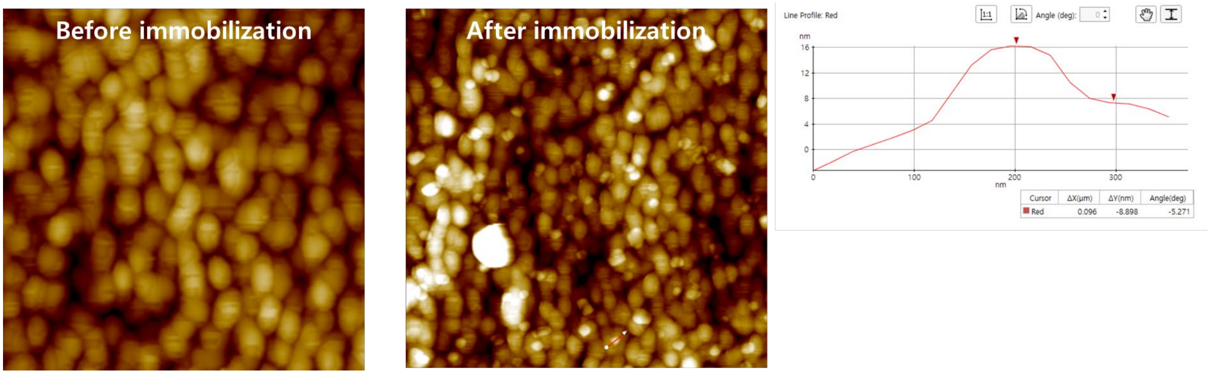

Figure S7. The AFM images of before and after AchE immobilization on PGFET surface.

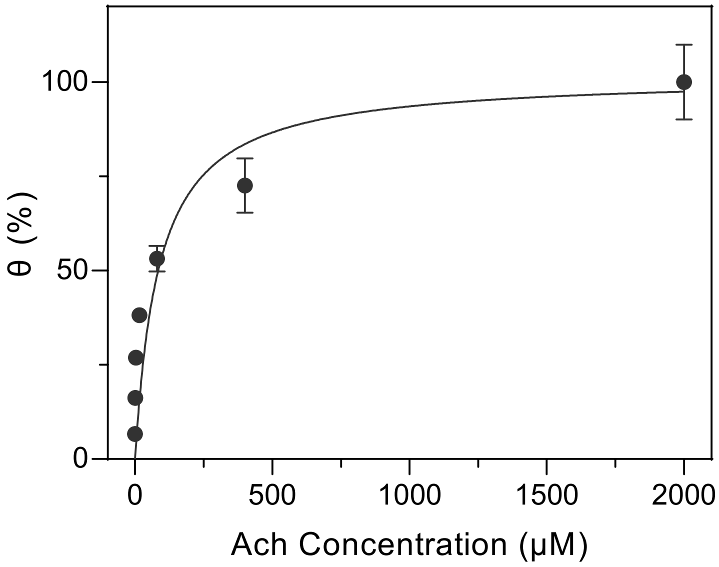

Figure S8. Fitting of the AchE activity to a Michaelis-Menten kinetics model.

The changes in $\Delta I_{ds}/I_0$ were also fitted to a thermodynamic model to investigate the enzymatic kinetics of AchE immobilized on the PGFET. The Hill equation is as follows:

$$\theta = \frac{[S]^n}{(K_D)^n + [S]^n}$$

where K_D is the dissociation constant, S is the substrate concentration, and n is the Hill coefficient, which describes the fractional saturation of the process. When n =1, the Hill equation equals Michaelis–Menten kinetics, the most well-known model for enzymatic reactions, and K_D equals the Michaelis–Menten constant (K_M), which is the concentration of substrate providing half of enzyme maximal activity. The quantified enzymatic responses of PGFET were fitted to Michaelis-Menten kinetics between θ and the concentration of Ach.

Table S1. Comparison of the analytical parameters of the PGFET and other recently reported biosensors for Ach detection.

Sensor type	Detection range (μM)	LOD (μM)	Sensitivity	Measurement setup	Comments	Ref.
rGO-FET	1-10000	1	13.9 mV/Ach dec	V_{ds}= 10mV, V_g = 1.0 to -1.0 V	Dirac point shift, static conditions	[1]
rGO-FET	100-10000	100	1.06 (ΔG/Gmin)/Ach dec	V_{ds}= 100 mV, V_g = -200 mV	Flow conditions	[2]
rGO-FET	5-1000	2.3	-850 μA/cm^2 Ach dec	V_{ds}= 100 mV, V_g = -200 mV	Flow conditions	[3]
Organic electrochemica l transistor	5-14	5	4.1 A/M cm^2	V_{ds}= -200 mV, V_g = 400 mV	Flow conditions	[4]
rGO-FET	0.108-2000	0.0723	1.34%/Ach dec (The relative changes in the source-drain current)	V_{ds}= 100 mV, V_g = -300 mV	Thermostatic, flow conditions	This work

References

1. Chae, M.-S.; Yoo, Y.K.; Kim, J.; Kim, T.G.; Hwang, K.S. Graphene-based enzyme-modified field-effect transistor biosensor for monitoring drug effects in Alzheimer's disease treatment. *Sens. Actuators B: Chem.* **2018,** *272*, 448–458.
2. Sohn, I.Y.; Kim, D.J.; Jung, J.H.; Yoon, O.J.; Thanh, T.N.; Quang, T.T.; Lee, N.E. pH sensing characteristics and biosensing application of solution-gated reduced graphene oxide field-effect transistors. *Biosens. Bioelectron.* **2013,** *45*, 70–76.
3. Fenoy, G.E.; Marmisolle, W.A.; Azzaroni, O.; Knoll, W. Acetylcholine biosensor based on the electrochemical functionalization of graphene field-effect transistors. *Biosens. Bioelectron.* **2020,** *148*, 111796.
4. Kergoat, L.; Piro, B.; Simon, D.T.; Pham, M.C.; Noel, V.; Berggren, M. Detection of glutamate and acetylcholine with organic electrochemical transistors based on conducting polymer/platinum nanoparticle composites. *Adv. Mater.* **2014,** *26*, 5658–5664.
